# Exosomes: Basic Biology and Technological Advancements Suggesting Their Potential as Ischemic Heart Disease Therapeutics

**DOI:** 10.3389/fphys.2018.01159

**Published:** 2018-11-19

**Authors:** Mayooran Shanmuganathan, Jeff Vughs, Michela Noseda, Costanza Emanueli

**Affiliations:** ^1^National Heart and Lung Institute, Imperial College London, London, United Kingdom; ^2^Royal Brompton and Harefield NHS Foundation Trust, London, United Kingdom; ^3^Bristol Heart Institute, University of Bristol, Bristol, United Kingdom

**Keywords:** exosomes, microRNAs, precision medicine, stem cells, synthetic biology, ischemic disease, angiogenesis, heart failure

## Abstract

Exosomes are small nano-sized vesicles that deliver biologically active RNA molecules and proteins to recipient cells through binding, fusion or endocytosis. There is emerging evidence that endogenous exosomes released by cardiovascular cells and progenitor cells impact cell survival and proliferation, thus regulating angiogenesis, cardiac protection and repair. These cardioprotective and regenerative traits have the potential to translate in to novel therapeutic options for post-ischaemic cardiac regeneration, thus potentially delaying the progression to ischaemic heart failure. Cellular stressors influence exosomes' secretion and the molecular composition of the exosome cargo, thus impacting on the above processes. Evidences are emerging that loading of proteins and RNAs in the exosomes cargos can be manipulated. Similarly, manipulation of exosomes surface proteins' expression to target exosomes to specific cells and tissues is doable. In addition, nature-inspired synthetic exosomes can be assembled to deliver specific clues to the recipient cells, including proliferative and differentiation stimuli, or shed paracrine signals enabling to reconstructing the heart homeostatic micro-environment. This review will describe exosome biogenesis and emerging evidence of exosome-mediated regenerative cell-to-cell communications and will conclude discussing possibilities of using exosomes to treat ischemic heart disease.

## Introduction

Ischemic heart disease (IHD) is the most frequent cause of heart failure (HF) (Kenchaiah et al., 2004). The early mortality rate after a myocardial infarct (MI) mortality has declined in the western world due the advent of clot-busting drugs and revascularization techniques including primary percutaneous coronary intervention (PPCI) and bypass surgery. Notwithstanding, the global burden of ischaemic HF continues to grow (Bui et al., 2011; Andersson and Vasan, 2018). This can be attributed to longer life expectancy and the increased prevalence of cardiovascular risk factors in young individuals (Bui et al., 2011; Andersson and Vasan, 2018). Development of proven pharmacological therapies for HF has stalled significantly in the last 2 decades, with the exception of Angiotensin Receptor—Neprilysin Inhibitor which was introduced in patients 3 years ago (McMurray et al., 2014). Mortality rate after 5 years from onset of HF symptoms remains high, at around 50% and heart transplantation represents the only definitive treatment currently available (Bui et al., 2011). After Orlic and Anversa published their infamous seminal paper reporting cardiac regeneration after bone marrow stem cell transplant to the mouse ischemic heart in 2001 (Orlic et al., 2001), stem cells have been eagerly pursued as the Holy Grail for post-ischemic regenerative medicine. Based on early human clinical trials, autologous cell therapy safety has been largely proven, although rescue function was showed minimal (Nguyen et al., 2016). Notwithstanding, there is a consensus that adult stem cell transplantation induces some positive effects that are mediated by paracrine actions (Hodgkinson et al., 2016). Exosomes are small extracellular vesicles (EVs) that have been proved to mediate cell-to-cell communications *in vitro*. Exosomes have been extracted from either conditioned cell culture media or biological fluids and then transplanted in animal disease models, showing to induce functional effects (Vicencio et al., 2015; Kalluri, 2016; Beltrami et al., 2017). In more details, exosomes released from cultured stem cells promoted angiogenesis and cardiac protection (Sahoo et al., 2011; Ibrahim et al., 2014). Therefore, exosomes might impersonate the leading characters in the paracrine play of stem cells, to the point that they have been proposed able to do “stem cells without the cells” (Hashimoto et al., 2018). On the other hand, it has been shown that exosomes are also involved in both beneficial and pathogenic cell-to-cell communication within the heart. Heart cells communicate with each other and with the blood and immune cells v*ia* exosomes and such communications are altered in diseased states, including MI and HF. These evidences further reinforce the quest for therapeutic exosomes to correct dysfunctional messengers, thus reinstating homeostatic conditions (Jung et al., 2017; Yang, 2018).

In this review article, we will explore the current understanding of exosome biogenesis, structure, contents and their possible roles in cardiac disease and as new therapeutic weapons to contrast ischemic HF. In this context, we will additionally discuss new approaches to both engineer endogenous exosomes and generate and design synthetic exosomes to deliver therapeutic materials to the heart.

## Myocardial infarction and the emerging role of exosomes

When a MI occurs, the blood flow to the heart decreases dramatically. The ischemic condition induces myocytes necrosis within 15–30 min with possible fatal consequences. Cells within and surrounding the infarcted area will be progressively lost due to necrosis and apoptosis. Cardiomyocytes, which are hugely dependent on oxygen for their active metabolism, are the first to display functional impairment such as contractile alterations and eventually die. Vascular cells will also be damaged. Later post-MI events encompass a combination of fibrotic, geometric, and hypertrophic changes associated with the development of HF through a combination of initially adaptive, and subsequently maladaptive ventricular remodeling responses (Sutton and Sharpe, 2000). Certain co-morbidities such as diabetes mellitus further worsen the clinical outcomes after MI, including by inducing microangiopathy (Iwakura et al., 2003; Prasad et al., 2005; Jensen et al., 2012; Lehrke and Marx, 2017). In the event of an established MI or severe angina, percutaneous or surgical intervention may restore blood flow to the subtended myocardium, but this does not usually improve clinical outcomes (Hochman et al., 2006) nor induce cardiac regeneration and reparative angiogenesis. Thus, there remains a need to find novel therapies to regenerate the infarcted myocardial tissue, restoring cardiac function, alleviating patients' symptoms and reducing mortality. Recent evidence shows that cardiac cells communicate via exosomes and that this communication system is altered in IHD (Arroyo et al., 2011; Chistiakov et al., 2016), particularly in diabetic subjects (Wang et al., 2014, 2016; Yuan et al., 2016; Ribeiro-Rodrigues et al., 2017; Li H. et al., 2018) This has stimulated more research in the role that these tiny vesicles may play as therapeutics (Emanueli et al., 2015; Marbán, 2018).

## Exosomes; biogenesis, structure and their cargo

Originated from the endosome or plasma membrane, EVs is a collective name of a heterogeneous family of membrane limited vesicles and consist of apoptotic bodies (sized 500 nm to-2 μm in diameter), microvesicles (100 nm−1 μm) and exosomes (30–150 nm; Kervadec et al., 2016). EVs were first thought to be a disposal of overabundant proteins (Trams et al., 1981). Today, EVs are recognized to be involved in mediating intracellular communication in normal and pathological processes (Trams et al., 1981; Johnstone et al., 1987; Minciacchi et al., 2015).

**Figure 1:**
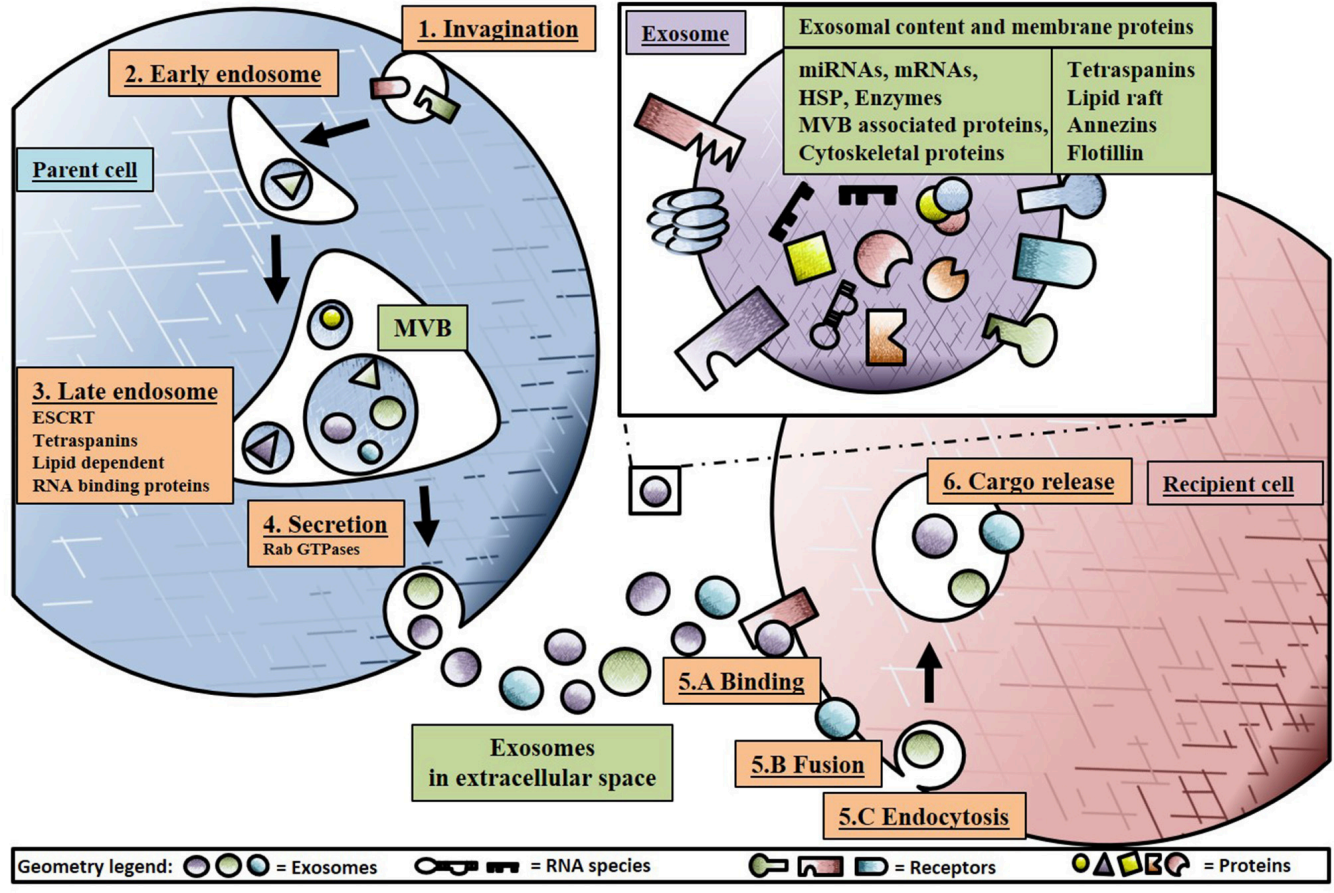
Schematic overview of exosome biogenesis and secretion. Starting at 1: Invagination of the plasma membrane, transporting intraluminal vesicles (ILVs) to 2. The early endosome fusing into multivesicular bodies (MVBs) 3. Formation of the late endosome loading exosomes with RNA cargo loading through Y-Box protein 1 (YBX1) and endosomal sorting complexes required for transport (ESCRT) and secreting 4. Through Rab GTPases into the extracellular space, homing exosome in to the recipient cell through 5.A Binding to receptors, 5.B Fusion with plasma membrane or 5.C Endocytosis with modulators such as extracellular signal-regulated kinase 1/2 (ERK1/2) and phosphoinositide 3-kinase (PI3K) pathways, 6. Releasing cargo in the intracellular space of the recipient cell inducing paracrine signaling effect.

The term “exosome” was coined by Rose Johnston in 1987 after discoveries a few years earlier that small 50–90 nm vesicles were released to the extracellular environment after fusion of late endosomes/multivesicular bodies (MVBs) with the plasma membrane (Johnstone et al., 1987). An overview of exosome biogenesis is provided in Figure 1. Exosome biogenesis starts with invagination of the plasma membrane, transporting the vesicle to the early endosome. Subsequently, the early endosome will mature into the late endosome, also known as MVB, through inward budding, generating, and accumulating intraluminal vesicles (ILVs) in the lumen of these organelles (Minciacchi et al., 2015). ILVs can be secreted as exosomes, but they can also be degraded or recycled within the parent cell. During ILV generation in MVBs, subsets of surface proteins such as D9, CD81, Alix, TSP-1, SOD-1, and pyruvate kinase aid in selectively sorting and loading of proteins, lipids and nucleic acids in to ILVs (Gupta et al., 2010). In addition, cargo sorting and loading of proteins are regulated by mechanisms such as endosomal sorting complexes required for transport (ESCRT) with subcomplexes 0, I and III. Additional ESCRT-independent mechanism include lipid dependent or tetraspanins with cluster of differentiation (CD) 81, 9, and 63 (Emanueli et al., 2015). The evolutionarily conserved late-domain (L-domain) pathway also contributes to the loading of proteins into exosomes. L-domains are used for the recruitment of ESCRT components to cell membranes and are required in MVB formation. As an example, the L-domain protein syntenin was identified in the recruitment of ALIX (an ESCRT-associated protein) and the subsequent formation and loading of exosomes (Baietti et al., 2012). Similarly, the L-domain-containing protein Ndfip1 has been identified in the spontaneous loading of proteins into exosomes (Putz et al., 2008, 2012) and even exploited to force the load of specific proteins into exosomes (Sterzenbach et al., 2017).

Exosomes contain different types of RNA molecules, including messenger RNA (mRNAs), circular RNA (circRNA), long non-coding RNA (lncRNAs), and microRNAs (miRs; Coumans et al., 2017; Li S. et al., 2018). miRNAs are small noncoding RNA capable of posttranscriptional gene expression regulation. To do that, each miR target a series of mRNAs, usually inducing mRNA degradation or translational inhibition. Exosomes protect their RNA cargos from RNase digestion (Arroyo et al., 2011; Vickers et al., 2011; Li Y. et al., 2015).

Importantly, exosomes shuttle biologically active miRs from their parent cell to recipient cells, thus spreading the miR regulatory actions (reviewed in Caporali et al., 2016). In fact, independently collected evidences suggest that upon exosome delivery of miRs to recipient cells, the miRs regulate gene expression in recipient cells, profoundly influencing cell behavior (Hergenreider et al., 2012; Bang et al., 2014; Deng et al., 2015; Beltrami et al., 2017; Mathiyalagan et al., 2017).

For understanding the mechanisms of miR loading into exosome, Shurtleff et al. investigated if certain miRs were specific and more abundant in exosomes instead of the host cells (HEK293T in this case). Interestingly, this was the case for miR-223 which was packaged by a RNA binding protein called Y-box Protein I (YBX1) (Shurtleff et al., 2016). Further investigation of this mechanism confirmed that YBX1 recognizes RNA molecules and is involved in export of miRs and other noncoding RNAs and transfer RNAs (t-RNAs; Shurtleff et al., 2016, 2017). However, this result was measured from HEK293 exosomes and the investigators stated that multiple RNA binding proteins could to be involved in the exosome packaging in different cell types (Villarroya-Beltri et al., 2013; Santangelo et al., 2016). It is thought that, similar to miRs, other RNA species such as circular RNA (circRNAs), messenger RNA (mRNA) and long non-coding RNA (lncRNAs), are also packaged in exosomes via regulated process, but these are still to be elucidated.

After cargo loading of exosomes, the MVB will fuse with the outer membrane, releasing the exosomes into the extracellular space through a mechanism involving Rab GTPases (Hsu et al., 2010). The exosomal membranes retain typology of the parent cell and contain lipid raft microdomains, aiding in recipient cellular uptake such as raft-mediated endocytosis (Zakharova et al., 2007). Subsequently, exosomes will reach recipient cells affecting their gene expression programme and in several cases also their function. Three types of internalization mechanisms have been described to be involved in exosome taking up by recipient cells: binding, fusion or endocytosis (Morrison et al., 2016). When exosomes bind to a recipient cell, they can act externally (i.e., without the need to be incorporated within the cell) as ligands to activate receptor mediated signal transduction. As an example, exosomes from tuberous sclerosis complex reportedly affected cells by activating Notch1 and mTOR, thus inhibiting differentiation of surrounding cells (Patel et al., 2016). When exosomes directly fuse with the recipient cell membrane the cargo is released into the cytoplasm (Parolini et al., 2009). Endocytosis mechanisms such as phagocytosis, micropinocytosis, clathrin-mediated, caveolin-mediated, and lipid raft-mediated endocytosis probably depend on cell type and their physiologic state but are not fully understood (Morelli et al., 2004; Fitzner et al., 2011; Nanbo et al., 2013; Mulcahy et al., 2014). Phagocytosis requires a subset of receptors depending on cell type before invagination. The process is also dependent on actin cytoskeleton, phosphoinositide 3-kinase (PI3K) and dynamin 2 (Feng et al., 2010). In micropinocytosis, the plasma membrane actively engulfs particles and is dependent on sodium and PI3K (Tian et al., 2014). Clathrin-mediated endocytosis, is aided by adaptor protein 2 and clathrin acting as a ligand and is observed in adrenal grand medulla tumor cells (Tian et al., 2014). In lipid raft endocytosis observed in tumor cells, extracellular signal-regulated kinase 1/2 (ERK1/2) signaling pathway is activated by exosomes and deregulates microRNAs that inhibit caveolin-1 leading to uptake of the exosome and transfer of its cargo (Svensson et al., 2013). After invagination, exosomes can act on signaling pathways releasing cargo through fusion, be degraded in the lysosome or recycled back to the plasma membrane. Interestingly, when heparin sulfate proteoglycans on the plasma membrane from the recipient cell are blocked with heparin or by adding scavenger receptor type B-1 removing cholesterol, there is a decrease in exosomes uptake (Atai et al., 2013; Christianson et al., 2013).

## The role of endogenous cardiac exosomes in cardiovascular disease

The adult human heart is made up of billions of cells and proximately a third of it is cardiomyocytes whilst the remainder is made up of endothelial cells (ECs), smooth muscle cells, neuronal cells, resident stem cells and fibroblasts (Zhou and Pu, 2016). Intercellular communication in both healthy and diseased states is very likely to be different and thus it is of crucial importance to study it in detail (Zhang et al., 2008; Barile et al., 2012; Hergenreider et al., 2012; Waldenström et al., 2012; Yu et al., 2012; Wang et al., 2014, 2016; Chistiakov et al., 2016; Garcia et al., 2016; Yang et al., 2016). Gupta and Knowlton were the first to describe the release of exosomes by cardiomyocytes from adult rats and observed that these exosomes contain heat shock protein 60 (HSP60) which can protect cells against injury such as myocardial infarction (Gupta and Knowlton, 2007). Waldenstrom and colleagues went on to show that EVs secreted by HL-1 cells (a cell model of cardiomyocytes) transported mRNA and that they were taken up by fibroblasts in a co-culture system, where they produced changes in gene expression in the recipient cells (Waldenström et al., 2012). The same group demonstrated that stimulation with TGF-β2 and PDGF-BB changed the RNA contents of the exosomes secreted by the HL-1 cells, thus giving credence to the idea that exosomes reflect the physiological state of the parent cells (Gennebäck et al., 2013). Similarly, Garcia et al. showed that when subjected to glucose deprivation *in-vitro*, neonatal rat cardiomyocytes release more exosomes. Interestingly, exosomes carry glucose, via glucose transporters, and glycolyic enzymes which are taken up by EC leading to increased glucose uptake, glycolytic activity and pyruvate production (Garcia et al., 2016). Yang et al. found that the serum exosomes of patients with acute MI were enriched with miR-30a. They also showed that exosomes from hypoxic cardiomyocytes release higher miR-30a in their exosomes and that this miR regulates autophagy in recipient cardiomyocytes (Yang et al., 2016). In another co-culture protocol, it was demonstrated that exosomes released by ECs were enriched with miR-143/145 and the miRs were transferred to smooth muscle cells, controlling their gene expression, thus to activate an atheroprotective programme (Hergenreider et al., 2012). Wang and colleagues found that exosomes released from cardiomyocytes impact EC proliferation, migration and angiogenesis *in vitro*. Interestingly, such responses were dramatically influenced by the origin of the cardiomyocytes: when they were cultured from healthy rats, exosomes promoted angiogenesis. By contrast, when the cardiomyocytes were prepared from diabetic rats, their exosomes promoted EC death and disrupted angiogenesis, possibly via transfer of miR-320 (Wang et al., 2014, 2016). Additionally, exosomes from cardiac myocytes subjected to cardiac pressure overload where shown to deliver functional Angiotensin II Type 1 Receptors (AT1R) to cardiomyocytes, skeletal myocytes, and mesenteric resistance vessels and were sufficient to confer blood pressure responsiveness to angiotensin II infusion in AT1R knockout mice (Pironti et al., 2015). Exosomes from hypoxic cardiomyocytes have been shown to regulate cell death in other cardiomyocytes (Zhang et al., 2008). Moreover, there is preliminary evidence that exosomes secreted from cardiomyocytes in acute MI contain TNF-alpha, a mediator of inflammation (Yu et al., 2012). Additionally, working on blood samples longitudinally collected from patients undergoing coronary artery-bypass-graft surgery using cardiopulmonary by-pass (“on-pump”) we provided the first in-man example of exosome trafficking out of the human heart (Emanueli et al., 2016). “On-pump” surgeries induce myocardial ischaemia/reperfusion injury. Working in this clinical setting, we provided evidences that the plasma concentrations of exosomes and their cargo of miRs of possible cardiac origin (miR-1, miR-24, miR-133a/b, miR-208a/b, miR-210) increased in the plasma on completion of surgery for up to 48 h. Importantly, the above responses were all positively correlated with changes in circulating high sensitive cardiac troponin, a gold standard laboratory biomarker of myocardial injury (Emanueli et al., 2016). These in-man data suggest the possibility that exosomes secretion by the stressed heart cells could play functional roles directing the heart response to surgery. Similar responses could contribute to post-MI HF.

Although in the above acute setting, a large percentage of circulating exosomes might have been of cardiac origin, under steady physiological and pathological states, plasma and serum exosomes originate are expected to originate from a variety of different cellular sources including the endothelium, platelets, and leucocytes. Circulating exosomes are thought to be of biological significance (Davidson et al., 2017, 2018) and could mediate post-MI responses (Vicencio et al., 2015).

The pericardial fluid, which is a plasma ultra-filtrate and surrounds the heart embedded in its pericardial sac, also contains exosomes, with a more probable cardiac origin, in comparison to peripheral plasma and serum (Beltrami et al., 2017). Exosomes from pericardial fluid contain clusterin, a glycoprotein able to improve myocardial performance through mediating epicardial activation, arteriogenesis and cardiomyocyte proliferation (Foglio et al., 2015). Additionally, pericardial fluid exosomes contain a highly proangiogenic miRNA: let-7b-5p and induce therapeutic angiogenesis *in vitro* and *in vivo* (Beltrami et al., 2017). It is legitimate to speculate that exosomes, via miRNAs and other mediators, play a role in cardiovascular cell-cell (paracrine) and distant (autocrine like) communication and this is affected by disease states (van Rooij and Olson, 2012).

## Exosomes as the mediators of stem cell therapy in ischaemic heart disease

**Figure 2:**
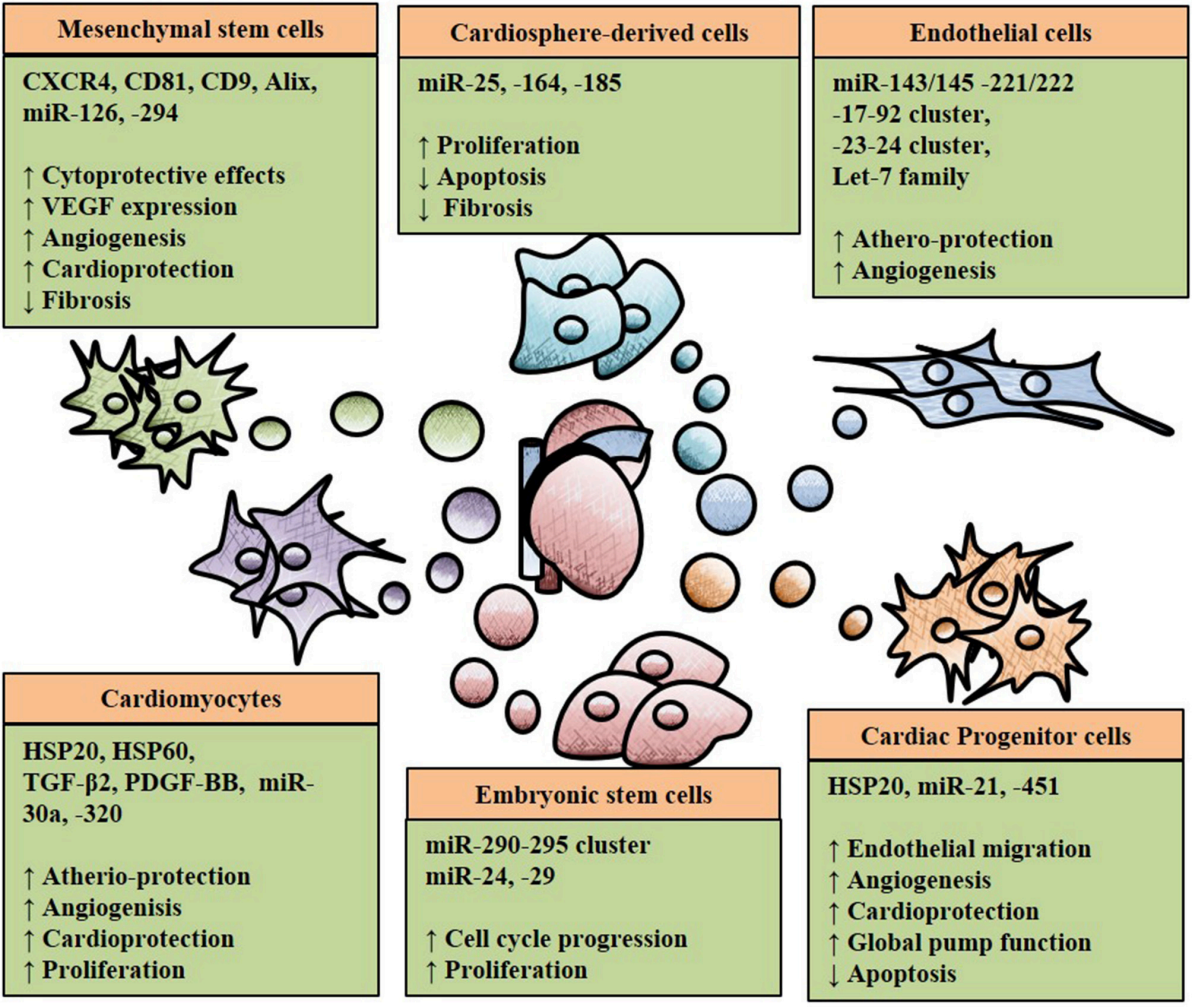
An overview of six different cell types secreting exosomes with therapeutic potential in ischemic heart disease. Exosomes represented as spherical geometries containing heatshock proteins (HSP), microRNAs (miRs), growth factors such as transforming growth factor beta 2 (TGF-β2). The impact of exosomes in inducing (↑) or decreasing (↓) is also indicated with the arrows.

Different types of stem cells (SC) and progenitor cells, such as mesenchymal SC (MSCs), embryonic SC (ESCs), hematopoietic SC and cardiac progenitors have shown the capability to differentiate toward cardiomyocytes (or at least cardiomyocyte-like cells) and vascular cells, at least *in vitro* (Cohn et al., 2000; Dixit and Katare, 2015; Noseda et al., 2015a). Moreover, as mentioned above, stem and progenitor cells support the survival of cardiovascular cells and angiogenesis responses by paracrine actions (Donndorf et al., 2013). In a quest for novel therapeutic solutions providing for cardioprotection, cardiomyogenesis and reparative angiogenesis, different types of stem and progenitor cells have been tested in animal models of MI, followed by first-in-man clinical trials, often on a small scale. Stem cell “therapies” have shown promises in the animal studies (Orlic et al., 2001; Miyahara et al., 2006; Chong et al., 2014; Noseda et al., 2015a). However, the results of the early clinical trials have been less exciting and they have rather stimulated a healthy scientific debate, which will be fundamental to the future advancements of this area of research. From several human and animal studies, it has become evident that, with few exceptions, the majority of the injected stem cells engrafted very poorly in the recipient heart and the rate of differentiation into myocytes and ECs was also limited *in vivo* (Balsam et al., 2004; Murry et al., 2004; Vrtovec et al., 2013; Kim et al., 2015; Noseda et al., 2015b). These evidences suggest that SC and progenitor cells exerted their benefit through hitherto unknown paracrine mechanisms (reviewed in Glembotski, 2017). In line with that, multiple scientific reports evidenced that MSCs, ESCs, CPSCs and induced pluripotent SCs (iPSC) mediated cardiac remodeling through paracrine signals (Lai et al., 2010; Chen et al., 2013; Khan et al., 2015; Noseda et al., 2015a; Wang et al., 2015; Kervadec et al., 2016). Intercellular communication between SCs and neighboring cells have been reported to induce angiogenesis and prevent apoptosis in cardiomyocytes by paracrine mechanisms (Lui et al., 2013; Xiao et al., 2016). Not all paracrine signals are soluble factors that can easily move through the extracellular environment to reach their targeted recipient cells before being degraded. However, a series of other messengers can be shuttled by exosomes and other EVs (Santangelo et al., 2016). As part of the paracrine activities of SC and other cells, EVs work as shuttle of miRs and other molecular form, conferring protection from degradation and helping their homing toward recipient cells. Multiple studies have shown that stem cell-derived exosomes induced protective and regenerative capabilities (Emanueli et al., 2015; Xu et al., 2017). Kang et al. reported that exosomes derived from CXCR4-overexpressing MSCs activated the Akt signaling pathway *in vitro* and in a murine MI-model (Kang et al., 2015). The *in vitro* results showed cytoprotective effects of exosomes on cardiomyoctes, inducing overexpression of VEGF and subsequently increasing vessel formation. *in vivo*, MSC sheets were pre-treated with exosomes and transferred on the infarcted area of the myocardium resulting in reduced infarct size by stimulation cell survival, improved cardiac remodeling and increased angiogenesis (Kang et al., 2015). Moreover, MSC-derived exosomes induced angiogenesis and aided athero-protective communications through miR-126 and miR-294, respectively (Vickers et al., 2011; Gonzalez-King et al., 2017). Additionally, Lai et al. identified the cardio protective effect of exosomes secreted from human ESCs-derived MSCs in an ischemia/reperfusion mouse model (Lai et al., 2010). Shao et al. identified exosomes derived from ESCs contain miR-24 and -29, aiding cardiac repair (Shao et al., 2017). miR-146-containing exosomes harvested from cardiospheres and injected in injured mouse hearts, inhibited apoptosis, promoted angiogenesis and cardiomyocyte proliferation, thus acting as critical steps for efficient cardiac protection and regeneration (Ibrahim et al., 2014). Sahoo et al. demonstrated fundamental evidence that exosomes secreted by bone marrow CD34(+) SCs promoted pro-angiogenic effects when tested *in vitro* and in a mouse model of ischemia and that their therapeutic effect was at least comparable with the one derived from transplantation of their parent cells (Sahoo et al., 2011; Bang et al., 2014). Later studies demonstrated that cardiac progenitor cells-derived exosomes play a role in post-MI cardiomyocyte survival *via* miR-21 and cardio protection *via* miR-451 (Chen et al., 2013; Xiao et al., 2016). Figure 2 presents an overview of SC-derived exosomes effects on the heart.

## Exosome-based therapies: harnessing endogenous and stem cell exosomes and engineering of synthetic exosomes

Exosomes' natural function to protect, target and deliver cellular components to recipient cells brings great interest to use them as novel tools for regenerative medicine. Moreover, it may be possible to artificially engineer exosomes (Arenaccio et al., 2018), package them with protective cargo including miRs, and deliver them to a patient with acute MI (to promote cardio-protection and therapeutic angiogenesis) and/or HF (to promote cardiac regeneration; Sluijter et al., 2018). It is hoped that exosomes will be perhaps more successful than previously attempts with stem cells (Wu et al., 2018). Using either naïve SC-derived exosomes or exosomes primed for specific drug loading are potential approaches to be considered. The first to show evidence of exosome-mediated delivery of nucleic acids was MJ Wood by injecting in mice exosomes containing small interference (si) RNA to knock down of BACE1, a therapeutic target in the setting of Alzheimer's disease (Alvarez-Erviti et al., 2011). Others, including ourselves, have published protocols to manipulate the exosome internal cargo (El-Andaloussi et al., 2012; Ong et al., 2014; Beltrami et al., 2017). Importantly, in clinical studies, exosomes have been already used as drug carriers promoting tumor rejection in patients (Rountree et al., 2011). Similarly, they were used in prostate cancer, lung cancer and in preclinical settings as anti-inflammatory agents and to inhibit a multi-drug resistant cancer by transporting the chemotherapeutic drug placlitaxel in mice (Morse et al., 2005; Rountree et al., 2011; Kim et al., 2016; Sun et al., 2016). Some promising technologies have emerged in cardiovascular exosome research such as direct programming of fibroblasts to cardiac myocytes. Tseliou et al. showed that intra-myocardially injected fibroblasts which had been primed with exosomes derived from cardiosphere-derived cells increased global pump function and vessel density while reducing scar mass in chronic MI mice model (Tseliou et al., 2015; Gallet et al., 2017). This effect could have been mediated at least in part by miR-146a (Ibrahim et al., 2014).

In order to improve the delivery of therapeutic messages to cardiac cells in need, exosomes can be engineered to modify their cargo and membranes. Vandergriff et al. conjugated exosomes with cardiac homing peptide to target cardiomyocytes *in vitro* and *in vivo* in ischemia/reperfusion animal model (Vandergriff et al., 2018). The promising results showed reduced infarct scar size and increased cellular proliferation and angiogenesis. Searching for the most suitable cardiac regenerative exosome cargo with cell-specific homing through peptide conjugation is still a challenge for cardiovascular disease (Kuehbacher et al., 2007). To obtain full regeneration with functional myocardium, the endothelial cells should be specifically targeted for blood vessel formation, the cardiomyocytes for proliferation and fibroblast for removing excessive scar tissue and aiding heart contraction (Gallet et al., 2017; Vandergriff et al., 2018). In this quest for holistic post-ischemic cardiac protection and regeneration, improved endogenous exosomes as well as nature-inspired synthetic exosomes could play fundamental roles. Therapeutic exosomes could be engineered with proangiogenic miRNAs such as miR-126 and/or let-7b-5p and with miR-146a (Bang et al., 2014; Ibrahim et al., 2014; Beltrami et al., 2017). In addition to miRs, the outer membrane of an exosome can be as important for protection as the cargo content itself. A study done by Vicencio et al. proved that plasma exosomes protect cardiomyocytes from hypoxia/reoxygenation injury through HSP70 located on the outer membrane of the exosome (Vicencio et al., 2015). Interestingly HSP70 binds to a toll like receptor on the recipient cell activating ERK p38MAPK pathway leading to HSP27, resulting in cardio protection. A possible engineered cardioprotective exosome could be enriched in its membrane for HSP70 and contain miR-146. Nakase et al. showed that in cancer cells, a pH-sensitive fusogenic peptide, GALA, together with ribosome inactivating protein saporin enhanced fusion with endosomal and exosomal membranes inside cells, increasing efficiency of target delivery (Nakase and Futaki, 2015). To translate such approach to the cardiovascular area, GALA and saponin could be combined with proangiogenic and cardiac protective exosomes, such as the one from bone marrow CD34+ cells and cardiospheres.

A series of recent technological advancements that could aid in harnessing **endogenous exosomes**. These include the possibility of pseudotyping exosomes for enhanced protein delivery in mammalian cells (Meyer et al., 2017). Viral pseudotyping is a strategy that has been used to create viral vectors with new tropism and trafficking properties. Pseudotyping manipulation of capsid proteins and envelope fusion glycoproteins are implicated in virus attachment and interactions with cellular receptors, determining cell tropism. Meyer et al. recently showed that a vesicular stomatitis virus (VSVG) glycoprotein can both load protein cargo onto exosomes and increase their delivery ability *via* a pseudotyping mechanism. In their hands, exosomes produced with the pseudotyping appeared good vehicles for the intracellular delivery of protein cargo, imparted by enhancing their intrinsic ability to deliver bioactive cargo to recipient cells (Meyer et al., 2017). Approaches to improve the loading of biologically active proteins into exosomes have also been developed. Those include exploiting the aforementioned L-domain pathway by Sterzenbach et al. (2017). Moreover, Yim et al. (2016), recently described a new exosome-based tool for intracellular delivery of target proteins: “Exosomes for protein loading via optically reversible protein–protein interactions” (EXPLORs). EXPLORs successfully loaded cargo proteins into newly generated exosomes by integrating a reversible protein–protein interaction module controlled by blue light with the endogenous process of exosome biogenesis (Yim et al., 2016). Importantly, treatment with protein-loaded EXPLORs increased intracellular levels of cargo proteins and their function in recipient cells *in vitro* and *in vivo*.

**Figure 3:**
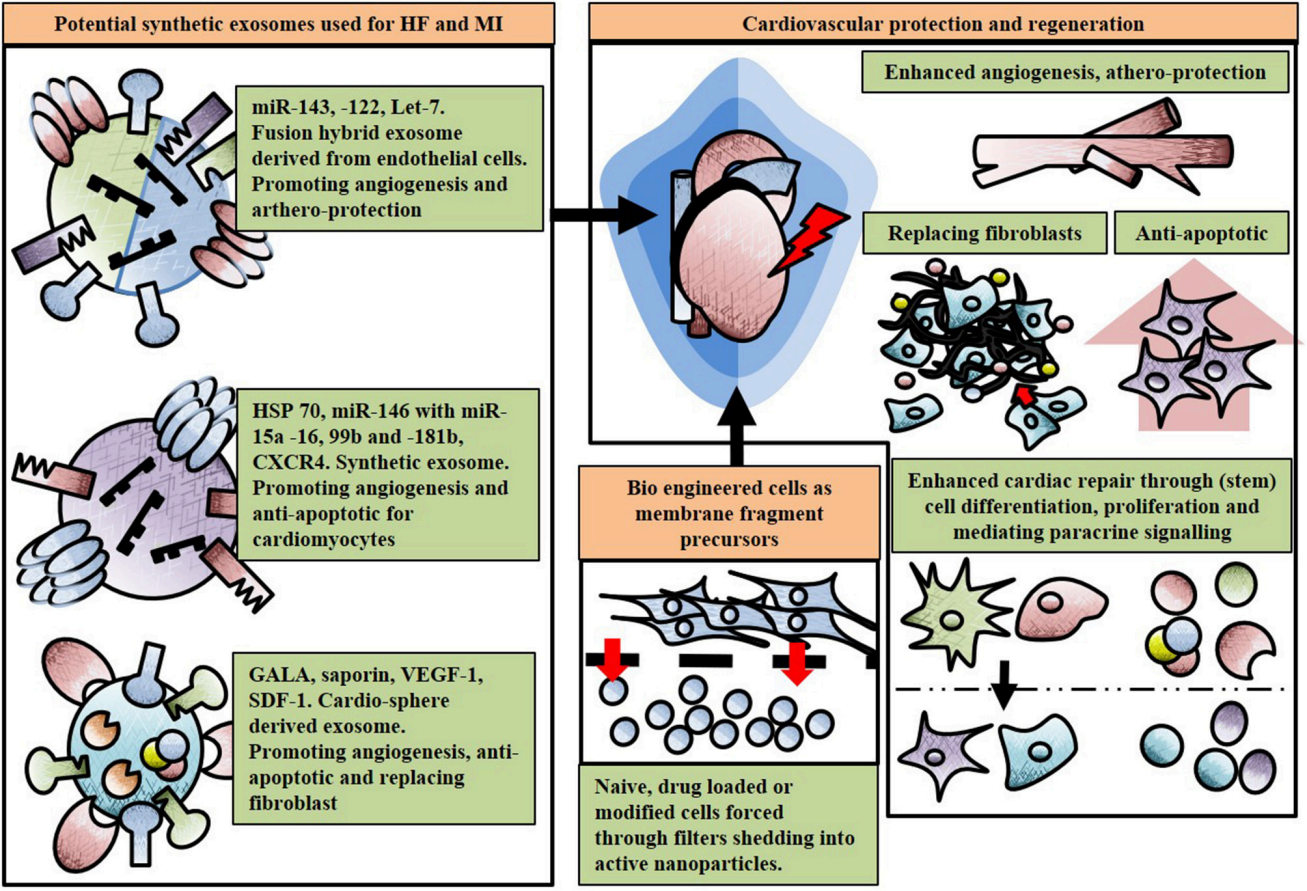
Potential use and effect of engineered and artificial exosomes. Exosomes can be enhanced through hybrid fusion, lipid conjugation or fragment precursors with various cargo and surface compositions for cardio-protection post myocardial infarction (MI) or cardiac regeneration in heart failure (HF). All non-cell geometries represent exosomes, growth factors such as vascular endothelial growth factor (VEGF), receptors, heat shock proteins (HSP) or microRNAs (miRs).

In designing naturally occurring exosomes or exosomes from modified cell populations as therapeutic tools, researchers face many hurdles with isolation, purification and production on a large scale and at a suitable clinical grade (Li S. P. et al., 2018). Thus, the development of bioinspired **fully synthetic exosomes** represents a new frontier in the so called “nanomedicine.” They should be robust and storable for prolonged period and therefore able to work as “off-the-shelf” therapies. In order to do this, we must first understand the (1) therapeutic cargo (e.g., protein, nucleic acids) that needs to be delivered to the diseased organ and (2) synthesize clinically effective synthetic exosomes containing this cargo (García-Manrique et al., 2018). There are multiple approaches for creating synthetic exosomes such as bio-engineering cells as membrane fragment precursors or through mimicking the plasma membrane by preparing artificial bilayers (García-Manrique et al., 2018). A novel example of the first approach was shown by Jang et al. who produced exosome-mimetic nanovesicles from a variety of cells with counter receptors such as LFA-1 to inhibit abnormal angiogenesis in murine cancer models (Jang et al., 2013). Both U937 and Raw 264.7 cells were loaded with chemotherapeutic agents such as doxorubicin, carboplatin and 5-fluorouracil, which were subsequently forced through 10-1 μm filters prior to centrifugation. These new particles maintained the topology of the plasma membrane and proved able to target tumors, reducing their size (Jang et al., 2013). An equivalent approach was used by Jeong et al. enhancing cell proliferation of murine skin fibroblast cells through particles produced *via* embryonic stem cell filtering (Jeong et al., 2014). With cardio-regeneration and protection in mind, this concept could be translated to obtain particles from cardiomyocytes, CPCs and ECs to enhance angiogenesis (Kuehbacher et al., 2007; Santangelo et al., 2016). As an example of the second of the aforementioned approaches, Li et al generated an efficient cargo loading mechanism by creating artificial biomimetic exosomes functioning as antigen presenting carriers to dendritic cells *in vivo* (Li K. et al., 2015). This method was performed by adding a water in oil micro-emulsion loaded protein with a micelle as outer lipid, and after evaporation of the water particles, the lipophilic group of the micelle would cover the inner membrane acting as a bi-layered membrane. This emulsion droplet greatly improved the capsulation efficiency of exosomes (Li K. et al., 2015). Interestingly, Sato et al. (2016) engineered hybrid exosomes derived from Raw 264.7 and CMS7 cells by membrane fusion with liposomes through a freeze-thaw method (Sato et al., 2016). The exosomes derived from Raw 264.7 cells and macrophage like cells contained high expression of HSP70 and tetraspanin and were fused in a 1:1 ratio with various lipid compositions (such as DOPC, DOPS, and DOTAP) through several cycles of freezing in liquid nitrogen and thawing at room temperature, rupturing and reconstruction both membranes. A similar approach was performed with CMS7 cells, murine fibro-sarcoma cells expressing HER2 receptor and CD63, with both hybrids successfully showing proof of principle through evaluation *via* western blot, flow cytometry and cellular uptake in HeLa cells with confocal laser scanning microscopy (Sato et al., 2016). This elegant method could potentially generate high specific exosome cargo containing a sub set of miR-143,−122, and let-7, aiding angiogenesis and athero-protection (Vickers et al., 2011; Emanueli et al., 2016; Sato et al., 2016; Beltrami et al., 2017). In addition, exosome fusion maintaining cargo from CPCs, CDCs, MSCs, ESCs, and cardiomyocytes with specific lipid homing composition could benefit anti-apoptosis of cardiomyocytes, reduce fibrosis, aid differentiation and proliferation after HF or MI. See Figure 3 for an overview of synthetic exosomes.

## Exosome homing

The role of integrins have been shown to play an important role in exosome cellular homing and indeed integrins have been even shown to guide homing of bioinspired synthetic exosomes (Ben-Arie et al., 2012). However, the exact mechanism and cell specific homing is not yet understood (Clayton et al., 2004). Using an *in vitro* binding assay, Denzer et al. has observed that exosomes isolated from B cell specifically bind to follicular dendritic cells (Denzer et al., 2000). Binding occurred through the major histocompatibillity complex class II (MHC class II), which is also on the surface of follicular dendritic cells. Additional homing molecules were equally as important such as costimulatory molecule CD86 and tetraspanin proteins CD37, CD53, and CD82, which interact with integrins and form oligomeric complexes with other tertaspanins (Denzer et al., 2000). Ohno et al. targeted xenograft breast cancer tissue expressing platelet-derived growth factor receptor fused with GE11 peptide with exosomes derived from human embryonic kidney 293 cells (Ohno et al., 2013). The results of *in vivo* studies in mice showed that the fluorescently labeled exosomes did target the cancer affected area, however was not highly specific due to immunogenicity. Exosomes have been engineered to target several receptors for therapeutic applications through peptide fusion with the N-terminus of exosome membrane proteins. However, these complexes can be degraded or cleaved as well, resulting in loss of homing capability (Xitong and Xiaorong, 2016). Interestingly, adding a glycosylation peptide motif and a small tag on the N-terminus of the peptides have been shown to protect from proteases which could be a potential counter measurement for these risks (Arnesen, 2011; Hung and Leonard, 2015; Xitong and Xiaorong, 2016). Table 1 summarizes and compares the properties of endogenous and exogenous exosomes.

**Table 1:** Comparison of endogenous and synthetic exosomes in ischemic heart disease based on current understanding.

**Trait**	**Endogenous**	**Synthetic**
**Exosome production**		
Quantity	High	High
Population	Heterogenous	Homogenous
Batch consistency	Medium	High
Harvest difficulty	Medium	Low
**Exosome controllability**	Semi cell specific	Highly cell specific
Cargo specificity	Non-specific	Cell specific
Cell homing	Semi specific	Highly specific
Drug loading	Feasible	Feasible
**Therapeutic use**		
Regenerative potency	High	Excellent
Disease specific	Medium/high	High
Adverse effects	Possible	Low probability
Personalized medicine potential	Medium	Excellent
Off-the-shelves potential	Low	High
Production cost	High	High at prototype level, but Low as exosome enter “mass production”

## Conclusions and future perspectives

Endogenous exosomes and laboratory engineered synthetic exosomes could bring a whole new era of therapeutic approaches for multiple diseases. The latter are particularly attractive. Such nature-inspired nanoparticles could represent paracrine cargo deliveries homing to their specific target location. In this review, we have discussed the role of endogenous exosomes in ischaemic heart disease as well as past attempts and new possibilities to engineered exosomes by manipulating their cargo with various types of molecules, such as miRNAs, proteins, peptides, and synthetic drugs. When designing exosomes, careful consideration of justifiable surface markers to express externally on the exosome membrane and cargo components has to be investigated to prevent possible off-target adverse effects. As discussed above, in the ischemic heart it is important to address the whole micro environment in a controlled matter. Hence, precision medicine approaches should consider the parallel and serial use of multiple exosome types to maximize the therapeutic responses. In ischemic heart disease, engineered exosomes could prove able to replace and surpass the first-generation stem cell therapies that have been shown to work via paracrine actions. Moreover, exosomes could play in concert with improved cell-based therapies and tissue engineering to deliver transformative therapeutic solutions.

## Author contributions

All authors listed have made a substantial, direct and intellectual contribution to the work, and approved it for publication.

### Conflict of interest statement

The authors declare that the research was conducted in the absence of any commercial or financial relationships that could be construed as a potential conflict of interest. The reviewer VB and handling Editor declared their shared affiliation.
